# The Role of Bispecific Antibodies in Relapsed Refractory Multiple Myeloma: A Systematic Review

**DOI:** 10.3390/antib12020038

**Published:** 2023-05-29

**Authors:** Razwana Khanam, Omer S. Ashruf, Syed Hamza Bin Waqar, Zunairah Shah, Saba Batool, Rameesha Mehreen, Pranali Pachika, Zinath Roksana, Mohammad Ebad Ur Rehman, Faiz Anwer

**Affiliations:** 1 Department of Hospital Medicine, Baystate Medical Center, Springfield, MA 01199, USA; 2 College of Medicine, Northeast Ohio Medical University, Rootstown, OH 44272, USA; oashruf@neomed.edu; 3 Downstate Medical Center, Department of Internal Medicine, State University of New York (SUNY), Brooklyn, NY 11203, USA; syed.waqar@downstate.edu; 4 Department of Internal Medicine, Louis A Weiss Memorial Hospital, Chicago, IL 60640, USA; zshah@weisshospital.com; 5 Department of Hospital Medicine, Unity Point Methodist Hospital, Peoria, IL 61636, USA; saba.m.batool@gmail.com; 6 Department of Internal Medicine, Jefferson Abington Hospital, Abington, PA 19001, USA; rameeshamehreen@gmail.com; 7 Department of Hematology-Oncology, University of Louisville, Louisville, KY 40202, USA; pachika.pranali@gmail.com; 8 Medical Officer, Sheikh Hasina National Institute of Burn and Plastic Surgery, Dhaka 1217, Bangladesh; zinathzuthy@gmail.com; 9 Department of Medicine, Rawalpindi Medical University, Rawalpindi 46000, Pakistan; ebadrehman.rehman@gmail.com; 10 Department of Hematology and Medical Oncology, Cleveland Clinic, Cleveland, OH 44195, USA

**Keywords:** bispecific antibodies, relapsed refractory multiple myeloma, teclistamab, elranatamb, talquetamab

## Abstract

Multiple myeloma is a heterogeneous clonal malignant plasma cell disorder, which remains incurable despite the therapeutic armamentarium’s evolution. Bispecific antibodies (BsAbs) can bind simultaneously to the CD3 T-cell receptor and tumor antigen of myeloma cells, causing cell lysis. This systematic review of phase I/II/III clinical trials aimed to analyze the efficacy and safety of BsAbs in relapsed refractory multiple myeloma (RRMM). A thorough literature search was performed using PubMed, Cochrane Library, EMBASE, and major conference abstracts. A total of 18 phase I/II/III studies, including 1283 patients, met the inclusion criteria. Among the B-cell maturation antigen (BCMA)-targeting agents across 13 studies, the overall response rate (ORR) ranged between 25% and 100%, with complete response/stringent complete response (CR/sCR) between 7 and 38%, very good partial response (VGPR) between 5 and 92%, and partial response (PR) between 5 and 14%. Among the non-BCMA-targeting agents across five studies, the ORR ranged between 60 and 100%, with CR/sCR seen in 19–63%, and VGPR in 21–65%. The common adverse events were cytokine release syndrome (17–82%), anemia (5–52%), neutropenia (12–75%), and thrombocytopenia (14–42%). BsAbs have shown promising efficacy against RRMM cohorts with a good safety profile. Upcoming phase II/III trials are much awaited, along with the study of other agents in concert with BsAbs to gauge response.

## 1. Introduction

Multiple myeloma (MM) is a heterogeneous clonal malignant plasma cell disorder accounting for approximately 15% of all annually reported hematologic malignancies in the Western world [1]. With the expanding arsenal of therapies, the 5-year relative survival of MM has improved to 57.9%, per Surveillance, Epidemiology, and End Results (SEER) Program 2012–2018 data [2].

Currently, different classes of myeloma therapies exist, including steroids, alkylators, proteasome inhibitors (PIs), immunomodulatory agents (IMiDs), selective inhibitors of nuclear export, monoclonal antibodies, and B-cell maturation antigen (BCMA)-targeted therapies; these agents are often used in combination for myeloma management [3]. However, myeloma cells may become resistant to the therapies owing to the tremendous pressure of the immunosuppressive bone marrow microenvironment (BMM) and genetic alteration within tumor cells, resulting in “relapsed and refractory” multiple myeloma (RRMM) [4].

Per the International Myeloma Working Group (IMWG), RRMM is defined as the progression of MM within 60 days of last therapy in patients who have achieved ≥ minimal response (MR) or those who are unresponsive to primary/salvage therapy [5,6]. MM patients undergo a variety of regimens, resulting in a heavily pretreated yet refractory and relapsed cohort “triple-refractory disease” defined as disease refractory to prior treatment with at least one anti-CD38 antibody, a PI, and an IMiD, and “penta-refractory disease” having prior exposure to two PIs, two IMiDs, and one anti-CD38 monoclonal antibody; the latter having overall survival of fewer than six months [7]. Chimeric antigen receptor T cells (CAR-T) are a newer addition to the treatment regimen of RRMM. These are fusion proteins that could be autologous or allogeneic depending on the source of origin [8]. The United States Food and Drug administration (FDA) has recently approved two genetically modified autologous CAR-T cells, namely idecabtagene vicleucel (March 2021) and ciltacabtagene autoleucel (February 2022) for use in RRMM after ≥4 prior lines of therapy including an IMiDs, a PI, and an anti-CD38 monoclonal antibody [9,10]. However, the incurable nature of the disease underscores the urgency of developing newer agents to treat this RRMM cohort effectively.

Bispecific antibodies (BsAbs) are artificially engineered antibodies that bind to two different antigens, whereas monoclonal antibodies bind to only one [11]. To facilitate an anti-tumor cytotoxic mechanism, one arm of BsAbs binds to CD3 molecules on tumor-specific T cells, and the other binds to an antigen on MM cells [12]. Some of the potential targets on MM cells are BCMA, G-protein-coupled receptor family C group 5 member D (GPRC5D), Fc receptor-homolog 5 (FcRH5), CD19, and CD38 [13]. BsAbs are also being developed against SLAMF7-and CD138 but are in therapeutic exploration phase [14]. This immunological synapse causes T-cell activation and degranulation, which perforate the MM cell membrane, thereby causing apoptosis by expressing perforin and granzyme B [15]. Interestingly, BsAbs activate T cells in a major histocompatibility complex 1 (MHC-1)-independent manner and do not require co-stimulation [16]. This reduces the risk of anergy in the absence of antigen-presenting cells (APCs) and cytokine signaling [17]. Moreover, it can evade tumor evasion by initiating tumor lysis even in low antigen expression levels [18].

## 2. Methods

We performed a literature search using three databases (PubMed, Cochrane Library, and EMBASE) following the Preferred Reporting Items for Systematic Reviews and Meta-analyses (PRISMA) guidelines. The MeSH terms and keywords used were ‘Multiple Myeloma’, ‘B Cell Maturation Antigen’, and ‘Bispecific Antibodies’ from inception to 31 October 2022. We also manually searched 64th American Society of Hematology (ASH) abstracts to look for updated results or new studies published on 15 November 2022. The search revealed 1182 articles imported to Endnote X9.3.3 citation management tool.

**Figure 1:**
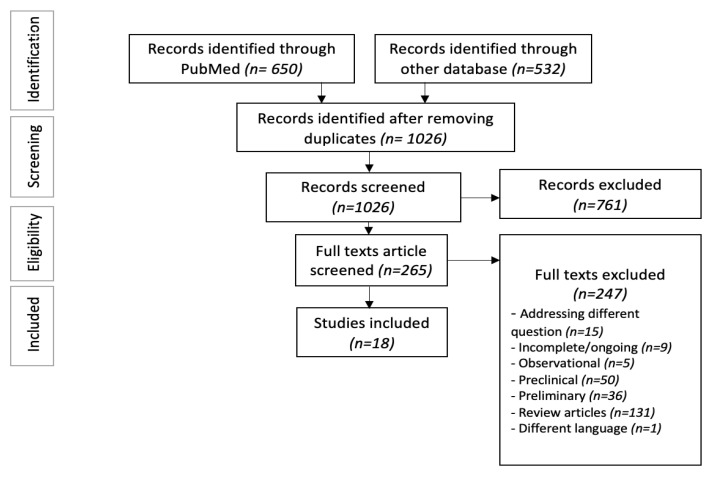
PRISMA flow diagram.

After removing 156 duplicates, two authors (SB and RM) independently screened the articles and eliminated 761 irrelevant studies after the primary screening. The secondary screening was performed by reviewing 265 full texts based on three predetermined inclusion criteria: (1) randomized or non-randomized clinical trials with results published, (2) age > 18, and (3) outcomes specific to RRMM patients. After excluding review articles, preclinical/preliminary studies, and observational studies, we included 18 clinical trials, reporting the outcomes of bispecific antibodies in RRMM patients (Figure 1) [19,20,21,22,23,24,25,26,27,28,29,30,31,32,33,34,35,36].

Two authors (PP and ZR) independently extracted data on patient demographics, treatment response, and treatment-related adverse events (TRAE). The treatment responses were expressed in the form of the overall response rate (ORR), complete response (CR), stringent complete response (sCR), very good partial response (VGPR), partial response (PR), stable disease (SD), and progression of disease (PD). The TRAE reported were cytopenia (anemia, neutropenia, thrombocytopenia, lymphopenia), cytokine release syndrome (CRS), neurotoxicity/immune cytokine-associated neurotoxicity syndrome (ICANS), overall infection, infusion related reaction (IRR), fever, fatigue, diarrhea, and transaminitis.

We used the National Institute of Health (NIH) quality assessment of systematic reviews and meta-analysis tool for our review and rated it as good [37]. Given the heterogeneity of the included studies, we were not able to perform any statistical analysis, but rather conducted narrative synthesis.

## 3. Results

Twelve studies were phase I, one study was phase II, four studies were phase I/II, and one was phase III. A total of 1283 patients were assessed, and the median age ranged from 62 to 68 (Table 1). The BsAbs that target BCMA are teclistamab, elranatamab, pacanalotamab, ABBV-383, REGN5458, pavuratamab, RO729089, and WVT078. On the other hand, the non-BCMA BsAbs that target tumor-associated antigens (TAAs) are talquetamab, RG6234, cevostamab, and ISB-1342. The efficacy and safety of 12 different bispecific agents are discussed in detail in Table 2, Table 3, Table 4 and Table 5.

Teclistamab, the first BsAb got approval of U.S. Food and Drug Administration (FDA) for use in adult RRMM patients who received at least four prior lines of therapy based on the data from the MajesTec-1 trial (NCT04557098). It targets BCMA and CD3 receptors. Touzeau et al. conducted a phase I/II trial (NCT04557098) evaluating safety and efficacy of teclistamab. A total of 38 patients were administered teclistamab subcutaneously at 1.5 mg/kg in weekly intervals. The ORR was observed in 40% (10/25) patients, with 20% (5/25) achieving >/= CR. Median time to first response was 1.2 months, and median time to best response was 2.1 months. [19]. In another phase I/II study (NCT03145181) conducted by Martinez-Lopez et al., 165 patients were administered teclistamab weekly subcutaneously at 1.5 mg/kg. The overall response rate (ORR) was 64% (105/165), with 30% (50/165) achieving CR or better [20]. A phase Ib trial (NCT04108195) assessed teclistamab in combination with daratumumab, an anti-CD38 antibody. A total of 46 patients were administered daratumumab subcutaneously at 1800 mg along with a 1.5–3 mg/kg subcutaneous injection of teclistamab either weekly or biweekly. The ORR was 78% (29/37), with 24% (9/37) achieving CR and 73% (27/37) VGPR. The median time to first response was 1 month [21]. Another phase 1b (NCT04722146) study investigated the combination of teclistamab along with daratumumab (1800 mg) and lenalidomide (25 mg), where teclistamab was given weekly at 0.72 or 1.5 mg/kg step-up dosing. The ORR was recorded in 81% (13/16) of patients at a 1.5 mg/kg dose. The median follow-up time was 4.17 months and median time to first response was 1 month [22]. In all four studies, neutropenia was the most common hematologic adverse event and CRS was the most common non-hematologic adverse event [19,20,21,22].

Elranatamab (PF-06863135) is a BCMA-CD3 BsAb. A phase I trial conducted by Rahe et al. evaluated the safety, pharmacokinetics, pharmacodynamics, and efficacy of elranatamab (NCT03269136). A total of 55 patients were administered the antibody subcutaneously at doses ranging from 80 to 1000 μg/kg on a weekly or biweekly basis. The ORR was 64% (35/55), with 38% (21/55) experiencing CR, and 56% (31/55) experiencing VGPR. The probability of being event-free for the responders was 59% [23]. A phase II trial (NCT04649359) conducted by Bahlis et al. recruited 123 RRMM patients. Patients were administered 76 mg elranatamab subcutaneously for a week. The ORR was 61% (75/123). Median time to response was 1.2 months [24]. A phase III (NCT05020236) study assessed the safety of elranatamab in combination with daratumumab, where elranatamab was given as a priming regimen for the first week followed by full weekly dose from cycle 1 to 6, and then biweekly dose from cycle 7. After a median treatment duration of 6.8 months, 50% of patients experienced CRS; median time to onset was 2 days. The study did not comment on the efficacy of the agent [25].

Pavuratamab (AMG 701) is a BCMA-CD3 half-life extended BsAb, which was evaluated in a phase I trial (NCT03287908) by Harrison et al. A total of 75 patients received weekly intravenous (IV) infusions of 0.8 mg step up dose prior to the target doses ≥1.2 mg to prevent severe CRS. At the 3–12 mg dosage, there was a 36% (16/45) overall response rate. At 12 mg, there was a 29% (2/7) response rate, with 14% achieving VGPR (1/7) and PR (1/7). The median time to response was 1 month, and time to best response was 2.8 months. CRS (61%) and anemia (43%) were the most common complications encountered [26].

AMG 420 (or pacanalotamab), a BCMA-CD3 BsAb, was evaluated in a phase I trial (NCT02514239) by Topp et al. A total of 42 patients were administered 0.2–800 μg/d of AMG-420 for up to 10 cycles spread over 6 weeks (4 weeks continuous, 2 weeks off treatment) intravenously. At the maximum tolerated dose of 400 μg/d, the ORR was 70% (7/10), with CR achieved in 50% (5/10) of patients: VGPR in 10% (1/10) and PR in 10% (1/10). However, 800 μg/d was not considered as a tolerable dosage, as two of the three patients experienced grade 3 CRS and grade 3 polyneuropathy, respectively [27].

RO7297089, a bispecific tetravalent antibody targeting BCMA and CD16a, was investigated by Plesner et al. (NCT04434469) in a phase I trial. A total of 21 patients were split into 5 groups of different dosages given as weekly IV infusions over 14-day cycles: 60 mg, 180 mg, 360 mg, 1080 mg, and 1850 mg. At a 1080 mg dose (*n* = 6), 17% (1/6) experienced PR, while 67% (4/6) experienced stable disease. The most common documented adverse event was anemia (52% [11/21]; 9 grade ≥ 3) [28].

ABBV-383, a BCMA-CD3 bispecific antibody, being studied in a phase I trial (NCT03933735). ABBV-383 was administered IV every 3 weeks over 1–2 h. Patients were then divided into 14 cohorts based on dose escalation (*n* = 81 dose escalation [0.025–120 mg]) and dose expansion (*n* = 51 for dose expansion [60 mg]). Median DOR was not reached, and median follow-up was 10.8 months. The ORR was 57% (69/122) with the following efficacies: 29% (35/122) CR/sCR, 14% (17/122) VGPR, and 14% (17/122) PR. A total of 57% experienced CRS (2% grade ≥ 3) and 37% experienced neutropenia (34% grade ≥ 3) [29].

REGN5458, a BCMA-CD3 bispecific antibody, being assessed in a phase I/II trial (NCT03761108) conducted by Bumma et al. A total of 167 patients followed a dose-escalation system. A step-up approach was utilized to minimize occurrence of CRS. The ORR was 52% (38/73) with CR achieved in 38% (27/73); greater response rate (75%) was noticed for those treated at ≥200 mg than for those treated with less than 200 mg (40.8%) [30].

WVT078, a BCMA-CD3 BsAb, investigated in a phase I trial (NCT04123418) by Raab et al. In this study, 33 patients were enrolled. Patients were administered WVT078 intravenously at 3, 6, 12, 24, 48, 64, 96, 192, and 250 μg/kg of body weight on a weekly basis. The ORR was 35% (9/26), with 12% (3/26) achieving CR. The ORR for 48–250 μg/kg dosages was 34.6% (9/26) with 12% (3/26) experiencing sCR/CR; clinical activity was evident at the 48 μg/kg [31].

Talquetamab is a GPRC5D-CD3 BsAb, the efficacy and safety of which are being assessed in a phase I/II study (NCT03399799/NCT04634552). Talquetamab was administered in 143 patients at 0.4 mg/kg subcutaneously on a weekly basis and 0.8 mg/kg subcutaneously biweekly. At a 0.4 mg/kg dose, the ORR was 73% (104/143), with 29% (41/143) achieving ≥CR and 58% (83/143) achieving ≥VGPR. The median progression-free survival was 7.5 months. A total of 45% had anemia, the most common hematological adverse event, and 79% CRS, the most common non-hematological adverse event [32]. In another phase Ib study (NCT04108195), talquetamab was administered along with daratumumab. A total of 46 patients were administered talquetamab at either 400 μg/kg weekly or 800 μg/kg biweekly with step-up dosing, along with 1800 mg daratumumab. At 800 μg/kg biweekly dose, the ORR was recorded 77% (17/22), with 27% (6/22) achieving ≥CR and 68% (15/22) achieving ≥VGPR. The median time to first response was 0.95 months. Anemia was recorded in 39% (20% grade 3/4) and CRS in 65% [33].

RG6234 is a G-protein-coupled receptor family C group 5 member D (GPRC5D) targeting BsAb, the pharmacokinetics, pharmacodynamics, and clinical activity of which is being assessed in a phase I trial (NCT04557150). A total of 51 patients received IV dose of 6–10,000 μg and 54 patients received subcutaneous (SC) dose of 30–7200 μg. In the IV cohort, the ORR was 71% (35/49), with CR/sCR and VGPR achieved in 29% (14/49) each, and PR in 14% (7/49). In the SC cohort, the ORR was 60% (29/48), with CR/sCR achieved in 19% (9/48), VGPR and PR achieved in 21% (10/48) each. CRS was the most observed adverse effect [34].

Cevostamab, a non-BCMA BsAb targeting FcRH5 and CD3, was studied in an ongoing phase I clinical trial (NCT03275103) by Lesokhin et al. Treatment was administered via IV infusion for 21 days per cycle, for a median of 17 cycles, with target range of 40–160 mg. The ORR was observed in 100% patients, with 63% (10/16) achieving CR/sCR, 31% (5/16) VGPR, and 6% (1/16) PR. A total of 19% (3/16) experienced PD and 19% (3/16) experienced SD. The only noted adverse event was overall infection, at a rate of 13% (2/16) [35].

ISB 1342, a non-BCMA CD3xCD38 BsAb, studied in a phase I trial to evaluate its safety (NCT03309111). Patients received ISB 1342 in 6 dose escalation groups from a 0.2/0.3 mg/kg to a 1.0/4.0 mg/kg dose intravenously, on a weekly basis. A total of 21% patients experienced anemia, and 42% experienced infusion-related reactions. No efficacies were recorded [36].

## 4. Discussion

In MM, the bone marrow microenvironment (BMM) is altered at a very early stage [38]. Furthermore, expression of surface marker BCMAis upregulated in MM, promoting tumor growth, immune evasion, and inhibiting apoptosis [39]. T-cell redirection has the propensity of eliminating MM cells, making BsAbs a next-generation therapy for this vulnerable population.

Our systematic review consisted of 18 phase I/II/III clinical trials with a sample size of 1283 [19,20,21,22,23,24,25,26,27,28,29,30,31,32,33,34,35,36]. Patients received 2–8 prior lines of therapy. Patients were exposed to different agents including anti-CD38 monoclonal antibody (mAb), daratumumab, elotuzumab, immunomodulatory agents (IMiDs), autologous stem cell transplant, protease inhibitors (PIs), and anti-BCMA agents, with at least 80% being triple refractory (818/1020) and 37% penta-refractory (256/700). Among the BCMA agents across 13 studies, the ORR ranged between 25% and 100%, with CR/sCR between 7 and 38%, VGPR between 5 and 92%, and PR between 5 and 14%. PD was seen in 12–67% patients, and SD in 30–56% patients. Regarding the non-BCMA agents, across five studies, the ORR ranged between 60% and 100%, with CR/sCR seen in 19–63%, VGPR in 21–65%, and PR in 6–21%. Per Lesokhin et al., PD seen in 19% patients (not recorded in the other four non-BCMA studies). The maximum response was seen secondary to cevostamab, and the combination of teclistamab, daratumumab and lenalidomide with teclistamab dosed at 0.72 mg/kg. The minimum response was seen after pavuratamab (AMG-701) administration. Across the 18 included studies, the median follow-up (MFU) varied between 1.7 and 12 months, and duration of response (DOR) ranged from 3.8 months to not reached to date. The decrease in the level of soluble BCMA (sBCMA) and serum-free light chain and conversely the increase in peripheral T-cell proliferation are the signs of treatment response [23,27,40]. Elranatamab and talquetamab were granted breakthrough designation by the FDA for adult RRMM patients based on the MagnetisMM-3 (NCT04649359) and MonumenTAL-1 (NCT04634552) trial, respectively [24,32].

The common adverse events seen across the included studies were CRS (17–82%), fever (8–39%), ICANS (0–9%), IRR (24–48%), infection (13–75%), anemia (5–52%), neutropenia (12–75%), lymphopenia (8–40%), thrombocytopenia (14–42%), transaminitis (12–30%), diarrhea (13–38%), fatigue (8–44%), etc. Death was observed in 10% patients (64 out of 633), but was not recorded in 11 studies; the etiologies were TRAE (*n* = 17), PD (*n* = 6), fulminant hepatitis (*n* = 1), acute respiratory distress syndrome/acute respiratory failure (*n* = 2), COVID-19 related (*n* = 1), sepsis (*n* = 2), retroperitoneal bleeding (*n* = 1), and subdural hematoma (*n* = 1). There was no reported treatment-associated death. Approximately 27% patients (228/836) discontinued treatment, secondary to TRAE (*n* = 43), PD (*n* = 159), death (*n* = 4), treatment completion (*n* = 3), physician decision (*n* = 6), consent withdrawal (*n* = 6), disease relapse (*n* = 2), symptomatic deterioration (*n* = 1), adverse event unrelated to drugs (*n* = 1), and anticancer therapy/surgery (*n* = 1). For some of the BsAbs, the maximum tolerated doses are stated in different clinical trials; for instance, 400 μg/day for pacanalotamab (AMG-420), 76 mg/week for elranatamab, 200 mg/week for REGN5458, and 40 mg once every three weeks for ABBV-383. The aforementioned doses may provide maximum efficacy with minimal side effects [24,27,29,30].

ISB 1442 is a fully human bispecific antibody with anti-CD38 and anti-CD47 arms, designed to overcome the resistance of daratumumab in RRMM patients. In vitro, it exhibited a higher killing potency in comparison to daratumumab and magrolimab [41]. Currently, a phase I/II clinical trial is underway investigating the efficacy of ISB1442 in RRMM (NCT05427812) [42]. In contrast, trispecific antibodies have garnered attention as novel immunotherapies capable of binding to three antigens simultaneously: two tumor cell antigens and one T-cell antigen, or one tumor antigen and two T-cell antigens, resulting in enhanced T-cell redirection and signal transduction, respectively [43]. Examples of trispecific antibodies include SAR442257 (CD38/CD3XCD28), HPN217 (BCMAXCD3), and CDR101 (BCMAXCD3XPD-L1). There are two phase I clinical trials studying the efficacy and safety of SAR442257 (NCT04401020) and HPN217 (NCT04184050) in RRMM, whereas data on CDR101 are still in the preclinical stage [44,45,46].

Our review has many limitations. Given the heterogeneity of the included studies, data could not be pooled to conduct a meta-analysis. Moreover, we could not compare outcomes between agents as patients received different prior lines of therapies, were either at fixed-duration-dose or dose-escalation cohorts, received monotherapy or combination therapy, had no control group, and had a variable follow-up period. Furthermore, cross comparisons of these trials should be given minimal weight, as these are mostly early phase (I/II) trials with different study population and drug escalation schemes. Herein, our review focused mainly on the relative efficacy of these agents for summary purposes and quick reference.

## 5. Conclusions

We conclude that BsAb has an excellent potential to emerge as an effective treatment for advanced RRMM; however, given aging T cells and changing repertoire throughout the disease course of myeloma, earlier introduction of BsAb during treatment before reaching the refractory stage might be of benefit. Given the selective nature of BsAb, adverse effects are also limited. The combined efficacy might result in a deeper hematological response, with early MRD negativity and less toxicity. The combination of different BsAbs together or with other drugs, such as daratumumab as an immunomodulator, are underway in ongoing trials and might be the future of treatment.

## Figures and Tables

**Table 1:** Demographics of RRMM patients treated with bispecific antibodies.

Agent Name	Target	Author, Year	Clinical Trial	Evaluable Patients/Sample Size	Median Age	Male Sex, %	Median Line of Treatment
Teclistamab [19]	BCMA-CD3	Touzeau et al., 2022	NCT04557098	38	63.5	63%	6
Teclistamab [20]	BCMA-CD3	Martinez-Lopez et al., 2022	NCT03145181	165	64	58%	5
Teclistamab+ Daratumumab [21]	BCMA-CD3+ anti-CD38	Otero et al., 2022	NCT04108195	46	67	48%	6
Teclistamab + Daratumumab + Lenalidomide [22]	BCMA-CD3+ anti-CD38+ IMiD	Searle at al., 2022	NCT04722146	32	62	87.5%	2
Elranatamab [23]	BCMA-CD3	Raje et al., 2022	NCT03269136	55	64	NR	5
Elranatamab [24]	BCMA-CD3	Bahlis et al., 2022	NCT04649359	123	68	55.3%	5
Elranatamab + Daratumumab [25]	BCMA-CD3+ anti-CD38	Grosicki et al., 2022	NCT05020236	28	68	NR	5
Pavuratamab (AMG-701) [26]	BCMA-CD3	Harrison et al., 2020	NCT03287908	75	63	NR	6
Pacanalotamab (AMG-420) [27]	BCMA-CD3	Topp et al., 2022	NCT02514239	42	65	64%	7
RO7297089 [28]	BCMA-CD16a	Plesner et al., 2021	NCT04434469	21	63	NR	8
ABBV-383 [29]	BCMA-CD3	D’Souza et al., 2022	NCT03933735	124	68	55%	5
REGN5458 [30]	BCMA-CD3	Bumma et al., 2022	NCT03761108	167	64	49%	6
WVT078 [31]	BCMA-CD3	Raab et al., 2022	NCT04123418	33	NR	NR	2
Talquetamab [32]	GPRC5D-CD3	Chari et al., 2022	NCT03399799/ NCT04634552	143	67	NR	5
Talquetamab+ Daratumumab [33]	GPRC5D-CD3+ anti-CD38	Donk et al., 2022	NCT04108195	46	65	52%	5
RG6234 [34]	GPRC5D-CD3	Carlo-Stella et al., 2022	NCT04557150	C1: 51 C2: 54	C1: 62 C2: 64	NR	C1:5 C2:4
Cevostamab [35]	FcRH5-CD3	Lesokhin et al., 2022	NCT03275103	16	66.5	NR	6
ISB 1342 [36]	CD38-CD3	Mohan et al., 2022	NCT03309111	24	67	63%	6

NR—not recorded, C1—cohort 1, C2—cohort 2, and IMiD—immunomodulatory agent.

**Table 2:** Efficacy of bispecific antibodies in RRMM.

Agent Name	Phase of Study	MFU, Months	ORR % (N)	CR/sCR, % (N)	VGPR	PR	PD	SD	DOR, Months
**BCMA agents**									
Teclistamab [19]	Phase I/II	6.9	40% (10/25)	20% (5/25)	NR	NR	NR	NR	NR *
Teclistamab [20]	Phase I/II	NR	64% (105/165)	30% (50/165)	NR	NR	NR	NR	NR *
Teclistamab + Daratumumab [21]	Phase Ib	7.2	78% (29/37)	24% (9/37)	73% (27/37)	NR	NR	NR	NR *
Teclistamab + Daratumumab + Lenalidomide [22]	Phase Ib	variable	C1: 100% (13/13) C2: 81% (13/16)	NR	C1: 92% (12/13) C2: NR	NR	NR	NR	NR
Elranatamab [23]	Phase I	12	64% (35/55)	38% (21/55)	56% (31/55)	NR	NR	NR	17.1
Elranatamab [24]	Phase II	6.8	61% (75/123)	NR	NR	NR	33% (40/123)	NR	NR *
Elranatamab + Daratumumab [25]	Phase III	NR	NR	NR	NR	NR	NR	NR	NR
Pavuratamab (AMG-701) [26]	Phase I	1.7	25% (17/69)	7% (5/69)	9% (6/69)	9% (6/69)	NR	NR	3.8
Pacanalotamab (AMG-420) [27]	Phase I	NR	31% (13/42)	21% (9/42)	5% (2/42)	5% (2/42)	60% (25/42)	NR	NR
RO7297089 [28]	Phase I	NR	NR	NR	NR	6% (1/18)	57% (12/21)	56% (10/18)	NR
ABBV-383 [29]	Phase I	10.8	57% (69/122)	29% (35/122)	14% (17/122)	14% (17/122)	12% (15/122)	30% (36/122)	NR *
REGN5458 [30]	Phase I/II	NR	52% (38/73)	38% (27/73)	NR	NR	NR	NR	NR *
WVT078 [31]	Phase I	NR	35% (9/26)	12% (3/26)	NR	NR	67% (22/33)	NR	NR
**Non-BCMA agents**									
Talquetamab [32]	Phase I/II	11	73% (104/143)	29% (41/143)	58% (83/143)	NR	NR	NR	9.3
Talquetamab+ Daratumumab [33]	Phase Ib	4	77% (26/34)	29% (10/34)	65% (22/34)	NR	NR	NR	NR *
RG6234 [34]	Phase I	C1: 7.1 C2: 3.9	C1: 71% (35/49) C2: 60% (29/48)	C1: 29% (14/49) C2: 19% (9/48)	C1: 29% (14/49) C2: 21% (10/48)	C1: 14% (7/49) C2: 21% (10/48)	NR	NR	C1: 12.9 C2: 8.8
Cevostamab [35]	Phase I	NR	100% (16/16)	63% (10/16)	31% (5/16)	6% (1/16)	19% (3/16)	NR	NR
ISB 1342 [36]	Phase I	NR	NR	NR	NR	NR	NR	NR	NR

NR—not recorded, NR *—not reached, C1—cohort 1, C2—cohort 2, MFU—median follow up, ORR—overall response rate, CR/sCR—complete response/stringent complete response, VGPR—very good partial response, PR—partial response, PD—progression of disease, SD—stable disease, and DOR—duration of response.

**Table 3:** Efficacy of bispecific antibodies arranged in descending order.

Agent	ORR	CR/sCR	VGPR	PR
Cevostamab [35]	100%	63%	31%	6%
Teclistamab + Daratumumab + Lenalidomide [22]	C1: 100% C2: 81%	NR	C1: 92% C2: NR	NR
Teclistamab + Daratumumab [21]	78%	24%	73%	NR
Talquetamab + Daratumumab [33]	77%	29%	65%	NR
Talquetamab [32]	73%	29%	58%	NR
RG6234 [34]	C1: 71% C2: 60%	C1: 29% C2: 19%	C1: 29% C2: 21%	C1: 14% C2: 21%
Elranatamab [23]	64%	38%	56%	NR
Teclistamab [20]	64%	30%	NR	NR
Elranatamab [24]	61%	NR	NR	NR
ABBV-383 [29]	57%	29%	14%	14%
REGN5458 [30]	52%	38%	NR	NR
Teclistamab [19]	40%	20%	NR	NR
WVT078 [31]	35%	12%	NR	NR
Pacanalotamab (AMG-420) [27]	31%	21%	5%	5%
Pavuratamb (AMG-701) [26]	25%	7%	9%	9%

NR—not recorded, ORR—overall response rate, CR/sCR—complete response/stringent complete response, VGPR—very good partial response, and PR—partial response.

**Table 4:** Toxicity of bispecific antibodies in RRMM.

Agent Name	CRS	Fever	Neurotoxicity/ICANS	Infusion Related Reactions	Overall Infection	Anemia	Neutropenia
Teclistamab [19]	63%	NR	3%	NR	42%	39%	55%
Teclistamab [20]	72%	NR	6%	NR	63%	50%	65%
Teclistamab + Daratumumab [21]	61%	NR	2%	NR	63%	46%	54%
Teclistamab + Daratumumab + Lenalidomide [22]	81%	25%	0	NR	75%	NR	75%
Elranatamab [23]	67%	NR	NR	NR	NR	NR	NR
Elranatamab [24]	58%	22%	3%	24%	62%	46%	43%
Elranatamab + Daratumumab [25]	50%	21%	0	NR	NR	NR	29%
Pavuratamab (AMG-701) [26]	61%	25%	8%	NR	NR	43%	23%
Pacanalotamab (AMG-420) [27]	38%	NR	5%	NR	33%	NR	NR
RO7297089 [28]	NR	NR	NR	48%	NR	52%	NR
ABBV-383 [29]	57%	19%	NR	NR	NR	29%	37%
REGN5458 [30]	48%	NR	NR	NR	NR	37%	29%
WVT078 [31]	61%	39%	NR	NR	NR	24%	12%
Talquetamab [32]	79%	NR	NR	NR	57%	45%	34%
Talquetamab + Daratumumab [33]	65%	NR	4%	NR	50%	39%	NR
RG6234 [34]	C1: 82% C2: 78%	NR	9%	NR	C1: 57% C2: 37%	C1: 14% C2: 5%	C1: 12% C2: 17%
Cevostamab [35]	NR	NR	NR	NR	13%	NR	NR
ISB 1342 [36]	17%	8%	NR	42%	NR	21%	NR

NR—not recorded, C1—cohort 1, and C2—cohort 2.

**Table 5:** Toxicity of the Bispecific Antibodies in RRMM.

Agent Name	Lymphopenia	Thrombocytopenia	Transaminitis	Diarrhea	Fatigue	Death	Treatment Discontinuation
Teclistamab [19]	40%	42%	NR	NR	NR	NR	NR
Teclistamab [20]	34%	38%	NR	NR	NR	NR	NR
Teclistamab + Daratumumab [21]	NR	33%	NR	33%	NR	NR	NR
Teclistamab + Daratumumab + Lenalidomide [22]	NR	NR	NR	38%	44%	3.1%	3.1%
Elranatamab [24]	26%	27%	NR	37%	33%	13.8%	NR
Elranatamab + Daratumumab [25]	NR	NR	NR	NR	NR	NR	0
Pavuratamab (AMG-701) [26]	NR	20%	NR	31%	25%	5%	NR
Pacanalotamab (AMG-420) [27]	NR	NR	12%	NR	NR	10%	95%
RO7297089 [28]	NR	19%	19%	NR	NR	19%	71%
ABBV-383 [29]	15%	23%	NR	27%	30%	27%	64%
REGN5458 [30]	NR	21%	NR	NR	34%	NR	3%
WVT078 [31]	18%	NR	30%	NR	NR	NR	76%
Talquetamab [32]	NR	27%	NR	NR	NR	0.7%	4.9%
Talquetamab + Daratumumab [33]	NR	35%	NR	NR	NR	NR	7%
RG6234 [34]	NR	C1: 14% C2: 19%	NR	NR	NR	C1: NR C2: 1.9%	C1: 3.9% C2: 3.7%
ISB 1342 [36]	8%	17%	NR	13%	8%	NR	NR

NR—not recorded, C1—cohort 1, and C2—cohort 2.

## Data Availability

No new data were created or analyzed in this study. Data sharing is not applicable to this article.
